# Serum Autoantibody Biomarkers for Management of Rheumatoid Arthritis Disease

**DOI:** 10.3390/bios13030381

**Published:** 2023-03-13

**Authors:** Esther Sánchez-Tirado, Lourdes Agüí, Marta Sánchez-Paniagua, Araceli González-Cortés, Beatriz López-Ruiz, Paloma Yáñez-Sedeño, José M. Pingarrón

**Affiliations:** 1 Department of Analytical Chemistry, Faculty of Chemistry, University Complutense of Madrid, 28040 Madrid, Spain; 2 Department of Chemistry in Pharmaceutical Sciences, Faculty of Pharmacy, University Complutense of Madrid, 28040 Madrid, Spain

**Keywords:** rheumatoid arthritis, immunosensing, electrochemical platform, magnetic microbeads, multiplex, autoantibodies

## Abstract

Rheumatoid arthritis (RA) is a systemic chronic autoimmune inflammatory disease that is characterized by the destruction of bone and production of autoantibodies such as rheumatoid factor (RF) and anticitrullinated protein antibodies (ACPAs). The high prevalence of this disease and the need of affordable tools for its early detection led us to prepare the first electrochemical immunoplatform for the simultaneous determination of four RA biomarkers, the autoantibodies: RF, anti-peptidyl-arginine deiminase enzyme (anti-PAD4), anti-cyclic citrullinated peptide (anti-CCP), and anti-citrullinated vimentin (anti-MCV). Functionalized magnetic beads (MBs) were used to immobilize the specific antigens, and sandwich-type immunoassays were implemented for the amperometric detection of the four autoantibodies, using the horseradish peroxidase (HRP)/H_2_O_2_/hydroquinone (HQ) system. The immunoplatform was applied to the determination of the biomarkers in human serum of twenty-two patients diagnosed with RA and four healthy individuals, and the results were validated against ELISA tests and the certified values.

## 1. Introduction

Among the chronic autoimmune diseases showing common pathogenic mechanisms, cytokine pathways and a systemic inflammatory cascade [1], rheumatoid arthritis (RA) is one of the most prevalent, affecting up to 0.46% of population worldwide [2]. This disease is characterized by damage in multiple joints and destruction of bone, leading to harsh disability and deterioration in the quality of life [3]. Its severity and the high prevalence have social and economic consequences, making the prevention of most serious effects necessary by starting the optimal treatment as early as possible [4]. To achieve the goal of prompt diagnosis, various disease-specific biomarkers have been proposed. Among them, anti-cyclic citrullinated protein antibody (anti-CCP) and/or immunoglobin M rheumatoid factor (RF) can be detected in blood in about three-quarters of patients [5]. However, neither of these tests are sufficiently specific for RA prognosis, which varies widely within seropositive and seronegative patient populations.

The calcium-dependent enzymes peptidylarginine deiminases (PADs) catalyze the conversion of amino acids–arginine residues to citrulline residues, and the resulting citrullinated peptides/proteins are the major component of antigenic determinants recognized by specific autoantibodies [6]. Among these enzymes, PAD4 is an important player in RA pathogenesis and the corresponding anti-PAD4 antibody is a highly specific biomarker for the disease. Furthermore, although citrullinated proteins are not used as RA biomarkers, the immune response against these proteins and the subsequent detection of the resulting autoantibodies (ACPAs) have been revealed to be of excellent diagnostic value for RA [7]. Particularly, anti-CCPs frequently appear before clinical manifestations of RA [8,9]. Regarding anti-MCV antibodies, their appearance is a consequence of vimentin secretion induced by TNF-α inflammatory cytokine. It has been shown that detection of these autoantibodies provides a comparable diagnostic value in RA to that of the anti-CCP assay, although their level seems to correlate closely with disease activity and therapeutic response in patients with RA. Evidence also exists highlighting the superiority of anti-MCV over anti-CCP in predicting joint erosions [10].

Current methods for the individual determination of autoantibodies as RA disease biomarkers use ELISA involving specific antigens for targets complexation. For example, the ELISA kit (AB178653, Abcam) for the determination of RF (https://www.abcam.com/rheumatoid-factor-igm-elisa-kit-ab178653.html, accessed on 10 February 2023) uses IgG Fc fragments to bind RF autoantibodies, followed by the addition of an HRP-conjugated anti-human IgM and colorimetric detection in the presence of TMB. A non-linear logarithmic calibration plot ranging between 3 and 300 IU mL^−1^ RF can be achieved, and the assay time is around 1 h 15 min. Similar strategies are involved in the ELISA kits for anti-PAD4. For instance, the kit from Cayman (PAD4 Autoantibody ELISA kit 500930; https://cdn.caymanchem.com/cdn/insert/500930.pdf, accessed on 10 February 2023) uses micro-wells coated with recombinant human PAD4 to bind the autoantibody and HRP-labeled goat-anti human Ig (H + L) as the detection immuno-reagent. The assay requires three hours at room temperature, and the linear calibration plot ranges between 15.6 and 1000 IU mL^−1^ anti-PAD4. ELISA kits for the determination of anti-MCV or anti-CCP use microwells coated with the antigens and similar sandwich-type immunoassays. For example, the protocol involved in the kit from MyBiosource for the determination of anti-MCV (MCVAb ELISA kit MBS9716536; https://cdn.mybiosource.com/tds/protocol_manuals/800000-9999999/MBS9716536.pdf, accessed on 10 February 2023) takes 1 h 30 min at 37 °C and allows a non-linear logarithmic calibration plot to be obtained between 5 and 80 μg L^−1^ anti-MCV with a LOD value of 0.5 μg L^−1^. A semi-logarithmic calibration plot from 20 to 1000 IU mL^−1^ anti-CCP is claimed for the ELISA kit from Demeditec (CCP Ab ELISA DE7760; https://www.demeditec.com/en/products/ccp-ab-elisa-de7760, accessed on 10 February 2023) in an assay time of about 1 h 5 min.

In a recent article [11], biosensors employed for the determination of biomarkers related to RA and osteoarthritis biomarkers were reviewed. However, among them, very few examples were reported related to the target autoantibodies analyzed in this work. An interdigitated microelectrode array was proposed by Chinnadayyala et al. [12] for the impedimetric detection of RF, using thioctic acid for covalent IgG Fc antigen immobilization. The method provided a linear calibration plot in the 1–200 IU mL^−1^ RF range and a limit of detection (LOD) of 0.6 IU mL^−1^. Another impedimetric design was also described for the label-free determination of RF in which YbYxOy sensing membranes were used for the immobilization of the autoantibody [13]. The biosensor provided a calibration range from 1.54 to 1.54 × 10^4^ IU mL^−1^. The determination of anti-MCV and anti-CCP antibodies and the advances in the use of citrullinated peptides/proteins for the diagnosis of autoimmune diseases have also been reviewed [14]. Electrochemical transduction involving multiwalled carbon nanotube-polystyrene nanocomposite was reported for the determination of anti-CCP, using a synthetic peptide covalently immobilized to the surface of the electrode [15]. More recently, a label-free impedimetric immunosensor for anti-CCP was reported by Chinnadayyala and Cho [16], which involved an interdigitated-chain-shaped microelectrode array modified with avidin-functionalized mercaptohexanoic acid for immobilization of a biotinylated synthetic peptide. The change in the charge-transfer resistance of the modified electrodes with the anti-CCP concentration was linear between 1 and 800 IU mL^−1^, with a limit of detection of 0.60 IU mL^−1^. The method was applied to spiked human serum samples.

The development of multianalyte biosensing strategies for the determination of disease biomarkers is an essential tool for achieving more valuable information than that provided by a single biosensor. Therefore, sensitive and cost-effective multiplexed bioassays are demanded for a fast and accurate clinical monitoring of autoimmune illnesses. In this context, the applications of paramagnetic iron oxide microparticles (MBs) in the clinical field have been extended due to their ability to be easily functionalized with diverse reactive moieties such as carboxyl, amine, or hydroxyl, as well as to interact with biomolecules (e.g., antibodies, peptides, or oligonucleotides) Therefore, MBs have been demonstrated to be a useful and versatile tool for the recognition of many target molecules [17]. Moreover, the magnetic nature of MBs allows us to enhance the analyte isolation and preconcentration from complex samples, thus simplifying the protocols for the preparation of the bioconjugates and providing a suitable methodology for the implementation of versatile magnetically assisted electrochemical platforms. These advantages, together with the availability of multiplexed screen-printed electrodes comprising several active surfaces, make the preparation of electrochemical biosensors that are suitable for the simultaneous determination of various biomarkers relatively easy.

In a previous work, our team developed an immunoplatform for the simultaneous determination of RF and anti-CCP which involved dual screen-printed carbon electrodes (SPCdEs) and functionalized MBs [18]. The implemented sandwich-type assays were applied to the determination of both targets in negative and positive RA controls. Although these target autoantibodies are the most frequently evaluated for RA diagnosis and monitoring, some patients are known to be seronegative for these biomarkers even though they suffer from RA. Therefore, the detection of other biomarkers with higher diagnostic efficiency is required [19]. Among them, anti-MCV and anti-PAD4 are recommended [8]. However, no biosensors for these autoantibodies have been reported so far.

In this paper, we report a magnetically assisted quadruple electrochemical immunoplatform for the fast, sensitive, and accurate simultaneous determination of the abovementioned four RA biomarkers: RF IgM, anti-PAD4, anti-CCP, and anti-MCV autoantibodies. With this contribution, we have expanded the recognition capacity of electrochemical platforms involving magnetic-assisted immunoassay to four biomarkers. In addition, we have demonstrated for the first time the possibility of determining together two recognized RA autoantibodies and two emergent biomarkers for which biosensors are not available so far. In addition, for the first time, we have demonstrated the analytical utility of the multiplexed immunoplatform by analyzing real human serum samples from 22 patients diagnosed with RA and 4 healthy patients (a total of 104 determinations). The obtained results were validated against ELISA tests.

## 2. Experimental

### 2.1. Apparatus and Electrodes

A CH1 1030B potentiostat (Chemical Instruments) equipped with the CH11030B software was used to obtain the amperometric measurements. The electrodes used were screen-printed carbon electrodes (SPCEs, DRP-110) with a single 4 mm Ø carbon working electrode (WE) and quadruple screen-printed carbon electrodes SPC4Es (DRP-4W110) consisting of four 2.95 mm Ø carbon WEs. These electrodes (Metrohm-Drop-Sens S.L.) include carbon auxiliary and Ag pseudo-reference electrodes and are provided with the specific DRP-CAC and DRP-CONNECT4W cable connectors. The spectrophotometric measurements for application of the ELISA methodologies were made using a Sunrise™ Tecan microplate reader with Magellan V 7.1 software. Homemade polymethyl-methacrylate (PMMA) casings with one or four Nd magnets (AIMAN GZ) embedded were used to introduce the SPCE or SPC4E with the captured bioconjugates into 10 mL glass electrochemical cells.

Other apparatuses used were a magnetic particles concentrator DynaMag^TM^-2 (Dynal Biotech ASA Thermo Fisher Scientific, Waltham, MA, USA), a Velp Scientific Vortex, a Thermo-shaker MT100 (Universal Labortechnik, Leipzig, Germany), a Crison model Basic 20+ pH-meter, an Elmasonic S-60 (Elma) ultrasonic bath, a Heidolph Reax Top homogenizer for small samples, and an MPW-65R centrifuge from MPW (Med. Instruments, Warszawa, Poland).

### 2.2. Reagents and Solutions

Carboxylic-functionalized magnetic microbeads (cMBs, 2.8 μm, 10 mg mL^−1^, Dynabeads^®^ M-270 carboxylic acid) were purchased from Dynal Biotech ASA (Thermo Fisher Scientific). Neutravidin-modified magnetic microparticles (Neutr-MBs, 1 μm Ø, 10 mg mL^−1^, SpeedBeads™) were from GE Healthcare. Human IgG Fc fragment (Fc(IgG), ab90285), rheumatoid factor IgM (RF, ab178653), and peroxidase-labeled anti-human IgM (HRP-IgM, ab178653) were from Abcam RF IgM ELISA kit (ab178653). PAD4 peptide (CAY-10500) and anti-PAD4 (human) standard antibody were from Cayman PAD4 Autoantibody ELISA kit CAY-500930. Biotinylated cyclic citrullinated peptide (CCP-biotin, orb55851) was from Biorbyt; human cyclic citrullinated peptide antibody (anti-CCP) and HRP-labeled anti-human IgG antibody (HRP-IgG) prepared in buffered serum matrix were from the ELISA kit DE7760 (Demeditec). MCV peptide (CAY-21942) was from Cayman, human anti-mutated citrullinated vimentin antibody (anti-MCV) was from the ELISA kit MBS9716536 (MyBiosource), and HRP-anti-IgG to human (ab97225) was from Abcam.

N-hydroxysulfosuccinimide (Sulfo-NHS), N-(3-dimethyl-aminopropyl)-N’-ethyl-carbodiimide (EDC), ethanolamine, hydroquinone (HQ), and hydrogen peroxide (H_2_O_2_, 30 % *v*/*v*) were from Sigma-Aldrich. Buffer solutions were 25 mM MES of pH 5.0 prepared from 2-(N-morpholine) ethano-sulfonic acid (Gerbu, Heidelberg, Germany); PBS, consisting of a 100 mM sodium phosphate buffer of pH 7.2 supplemented with 0.0920 g NaCl (Labkem, Barcelona, Spain) and 0.0020 g KCl (Scharlau, Barcelona, Spain) in 100 mL deionized water; PBST, prepared by addition of 0.100 g Tween 20 (Aldrich, MO, USA) to 100 mL PBS; 100 mM PB of pH 8.0; 50 mM PB of pH 6.0; and commercial casein blocking buffer in PBS (BB) from Thermo Scientific were also used. Deionized water was from a Millipore Milli-Q purification system (18.2 MΩ cm).

### 2.3. Samples

The analyzed samples were purchased in Central BioHub^®^ GmbH, Hennigsdorf, Germany, and consisted of human serum from 22 patients diagnosed with RA and 4 healthy individuals. Certified concentrations of RF, anti-MCV, and/or anti-CCP for several of the samples were provided by Central BioHub^®^, but the anti-PAD4 concentration was unknown. The four biomarkers were determined with the immunoplatform in each serum sample without prior treatment, except for an 1/1000 dilution for anti-PAD4, making a total of 104 determinations. In addition, the obtained results were compared with those provided by the corresponding individual ELISA test and the certified contents informed by the supplier.

### 2.4. Procedures

The procedures followed for the preparation of the magnetic immunoconjugates are described in the Supplementary Materials.

#### 2.4.1. Amperometric Detection

A total of 10 µL of the corresponding immunoconjugates (HRP-IgM-RF-Fc(IgG)-cMBs, HRP-anti-IgG-anti-PAD4-PAD4-cMBs, HRP-anti-IgG-anti-MCV-MCV-cMBs, or HRP-IgG-CCPA-CCP-biotin-Neutr-MBs) was suspended in 50 mM PB of pH 6.0 and magnetically captured on the respective WE surface of the SPCE or SPC4E placed in the PMMA housing with the embedded neodymium magnets. Then the single or quadruple determinations were performed by immersion of the casing/electrode assembly with the immunoconjugates into an electrochemical cell containing 20 mL of 50 mM PB of pH 6.0 and 200 µL of 0.1 M HQ solution prepared in the same buffer and by applying a detection potential of −0.20 V vs. Ag pseudo-reference electrode. Whenever the background current was stabilized (approximately 100 s), 100 μL of a recently prepared 0.1 M H_2_O_2_ solution in 50 mM PB of pH 6.0 was added to the cell, and the current variation at each electrode produced by the enzymatic reduction of H_2_O_2_ mediated by HQ was recorded until the steady state was reached. The amperometric responses were measured as the difference between the steady state and the background currents, and the given values were the mean of three replicates, with the error bars estimated as the triple of standard deviation of each set of replicas (α = 0.05).

#### 2.4.2. Determination of RF, Anti-PAD4, Anti-MCV, and Anti-CCP in Human Serum

The simultaneous determination of the four RA biomarkers in the human serum of patients diagnosed with RA, as well as of healthy individuals, was performed by applying the procedure described above to 25 µL aliquots of undiluted sample (RF, anti-MCV, and anti-CCP) or 1/1000 diluted sample (anti-PAD4) in 50 mM PB of pH 6.0. After the amperometric measurements, the current responses were interpolated into the calibration plots constructed with the respective standard solutions.

## 3. Results and Discussion

**Figure 1:**
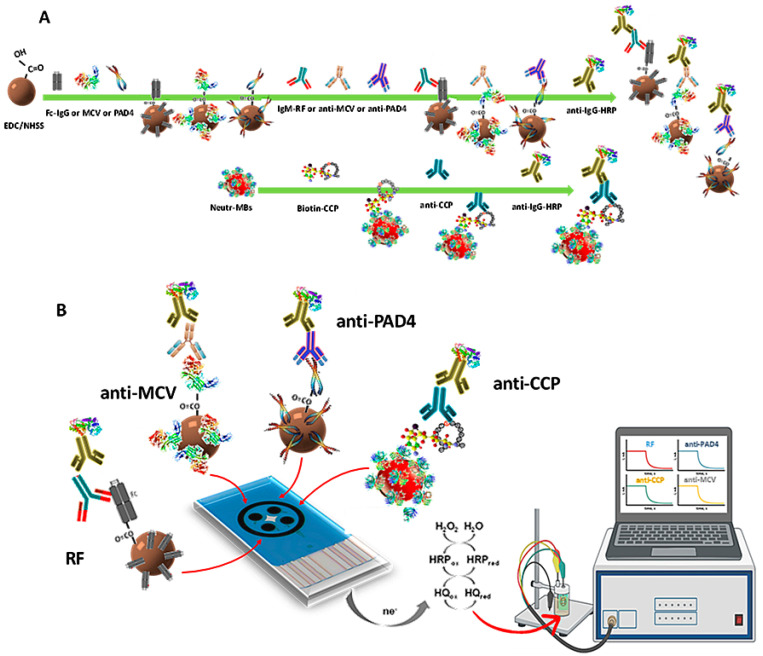
(**A**) Scheme of the steps for the preparation of HRP-IgM-RF-Fc(IgG)-cMBs, HRP-IgG-anti-PAD4-PAD4-cMBs, HRP-IgG-anti-MCV-MCV-cMBs, and HRP-IgG-anti-CCP-CCP-Biotin-Neutr-MBs magnetic immunoconjugates and (**B**) their capture on the SPC4E, as well as the reactions involved in the simultaneous amperometric detection.

The first immunoplatform suitable for the simultaneous determination of four RA autoantibodies biomarkers (RF, anti-PAD4, anti-MCV, and anti-CCP) was prepared in this work according to a systematic and relatively simple way. Magnetic bioconjugates onto functionalized MBs were prepared by covalent immobilization of the respective antigens on cMBs (Fc(IgG), PAD4, and MCV) or by affinity interaction on Neutr-MBs (CCP-biotin). After binding with the corresponding target autoantibody, the respective sandwich-type configurations were accomplished using a secondary antibody labeled with HRP (HRP-IgG, except HRP-IgM for RF). Figure 1 shows a scheme of (A) the steps involved in the preparation of the magnetic immunoconjugates; and (B) the quadruple immunoplatform, where, from left to right, HRP-IgM-RF-Fc(IgG)-cMBs, HRP-IgG-anti-PAD4-PAD4-cMBs, HRP-IgG-anti-MCV-MCV-cMBs, and HRP-IgG-anti-CCP-CCP-Biotin-Neutr-MBs magnetic immunoconjugates were captured on each WE of the SPC4E. The amperometric currents provided by the reduction of H_2_O_2_ in the presence of HRP and mediated by HQ were simultaneously measured, as shown in the illustrative display, with each one being proportional to the concentration of a target biomarker.

### 3.1. Optimization of the Experimental Variables

The variables involved in the preparation and functioning of the immunoplatform were optimized by individually testing their effect on the amperometric response for each target autoantibody. The selected value for each variable was taken according to the criterion of a larger specific-to-unspecific current (S/N) ratio. The obtained results are described in Supplementary Materials Figures S1–S4 and summarized in Table 1.

Other experimental conditions, such as the amount of functionalized MBs, the composition of the H_2_O_2_/HQ system, or the detection potential, were optimized in previous works [20,21]. As recommended by the application protocols of carboxylated MBs (https://www.thermofisher.com/es/es/home/references/protocols/proteins-expression-isolation-and-analysis/protein-isolation-protocol/m-270-carboxylic-acid.html, accessed on 10 February 2023) in the immunoplatforms for anti-PAD4 and anti-MCV, 1M ethanol-amine solution prepared in 0.1 M PB of pH 8.0 was used to block the remaining active unreacted groups of cMBs. This blocking step was not necessary in the case of the RF since, as described in the Supplementary Materials, very low non-specific responses were observed probably due to the high specificity of the Fc(IgG)-cMBs conjugate for the RF antibody. Furthermore, the significantly low unspecific currents observed in the preparation of the anti-CCP immunoplatform can be attributed to the use of Neutr-MBs since the nearly neutral charge of neutravidin minimizes non-specific interactions conversely to that observed when working with streptavidin- or avidin-functionalized MBs [22].

### 3.2. Analytical Characteristics of the Immunoplatform for the Determination of the Four Antibody Biomarkers

**Figure 2:**
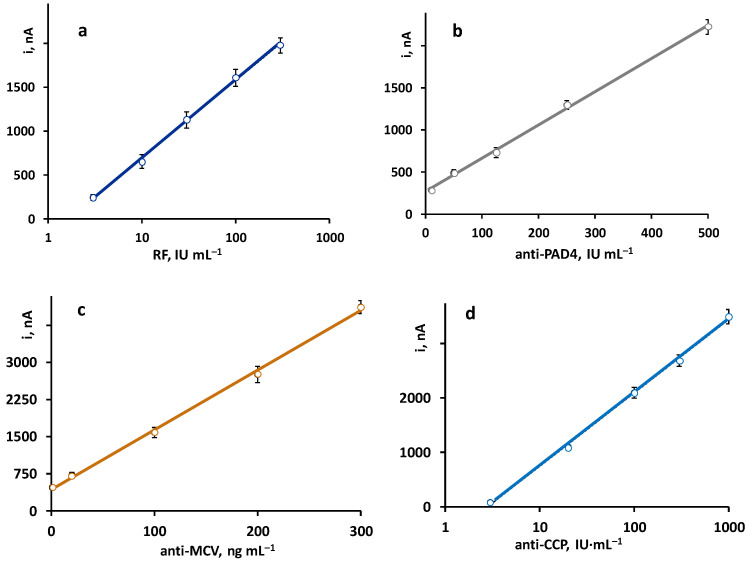
Calibration plots obtained with the quadruple immunoplatform for the simultaneous determination of (**a**) RF, (**b**) anti-PAD4, (**c**) anti-MCV, and (**d**) anti-CCP antibodies.

The calibration plots constructed with the quadruple platform, using the optimized working conditions for each autoantibody, are shown in Figure 2. As expected, according to the sandwich-type configuration of the immunoassays, the recorded currents were directly proportional to the concentration of each antibody. Linear ranges for anti-PAD4 and anti-MCV and semilogarithmic plots for RF and anti-CCP were obtained with the parameters summarized in Table 2.

The achieved analytical characteristics were compared with those claimed for the ELISA kits involving the same immunoreagents (https://www.abcam.com/rheumatoid-factor-igm-elisa-kit-ab178653.html, accessed on 10 February 2023; https://cdn.caymanchem.com/cdn/insert/500930.pdf, accessed on 10 February 2023; https://cdn.mybiosource.com/tds/protocol_manuals/800000-9999999/MBS9716536.pdf, accessed on 10 February 2023; and https://www.demeditec.com/en/products/ccp-ab-elisa-de7760, accessed on 10 February 2023). Although, in general, similar dynamic ranges and LOD values were observed, some advantages of the bioplatform should be highlighted. For instance, the semilogarithmic calibration graph for RF is linear contrary to the non-linear range provided by the ELISA kit. Regarding anti-MCV, the calibration plot exhibits a wider linear range (1–300 ng mL^−1^ vs. 5–80 ng mL^−1^), while, in the case of anti-PAD4, a slightly lower LOD value was obtained (5.5 IU mL^−1^ vs. 15.6 IU mL^−1^). Regarding precision, all the RSD values obtained with the immunoplatform are equal or below 5%, which, for example, are significantly lower than those claimed for the anti-MCV ELISA test, with intra- and inter-assay precision values of 9% and 11%, respectively. In addition, the assay test times are considerably shorter. For instance, in the case of anti-PAD4, the ELISA assay requires 3 h and 10 min, while the single amperometric determination takes 1 h and 15 min.

On the other hand, the immunoplatform provides similar dynamic ranges and LOD values than those reported for the few electrochemical biosensors existing in the literature for the single determination of RF [12,13] and anti-CCP [15,16] and for the dual determination of both targets [18]. Obviously, the main advantage of the developed immune platform is the possibility of performing the simultaneous determination of the four RA biomarkers in an assay time of about 2 h counting since the immobilization of the antigens, as well as using a low sample volume of 25 mL for each target.

The storage stability of the antigen–MB bioconjugates was evaluated by keeping some of them prepared in the same day at 8 °C in 50 μL PBS (pH 7.2) and measuring during various days the amperometric currents provided by the immunoplatforms constructed from the stored conjugates. The results displayed in Supplementary Figure S5 show that the responses obtained each control day remained within the control limits set at ±2 s, where s is the standard deviation of the measurements (*n* = 3) carried out the first day of the study for at least 20 days.

### 3.3. Selectivity

**Figure 3:**
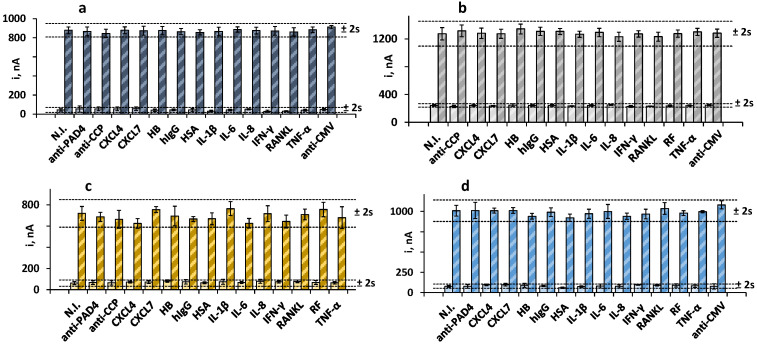
Amperometric measurements obtained with the quadruple bioplatform for 0 (light bars) and (**a**) 30 IU mL^−1^ RF (dark blue); (**b**) 250 IU mL^−1^ anti-PAD4 (dark gray); (**c**) 20 IU mL^−1^ anti-MCV (dark yellow), or (**d**) 20 IU mL^−1^ anti-CCP (blue) prepared in the absence and in the presence of 100 IU mL^−1^ RF (**b**–**d**); 250 IU mL^−1^ anti-PAD4 (**a**,**c**,**d**); 40 ng mL^−1^ anti-MCV (**a**,**b**,**d**), 100 IU mL^−1^ anti-CCP (**a**–**c**), and 50 ng mL^−1^ CXCL4, 50 ng mL^−1^ CXCL7, 5 ng mL^−1^ HB, 1 mg mL^−1^ hIgG, 50 pg mL^−1^ HSA, 50 pg mL^−1^ IL-1β, 300 pg mL^−1^ IL-6, 300 pg mL^−1^ IL-8, 100 pg mL^−1^ IFN-γ, 50 pg mL^−1^ RANKL, 100 IU mL^−1^ RF, or 200 pg mL^−1^ TNF-α. Error bars are estimated as three times the standard deviation value of three replicates.

We tested for the presence of other antibodies and proteins that may coexist with the target biomarkers in human serum and affect the electrochemical responses of the immunoplatform. To do that, the amperometric currents in the absence and in the presence of 30 IU mL^−1^ RF, 250 IU mL^−1^ anti-PAD4, 20 IU mL^−1^ anti-CCP, and 20 ng mL^−1^ anti-MCV were measured and compared with the current values measured in solutions containing the potential interferents at the expected concentrations in the serum of healthy individuals. Figure 3 shows that no significant interference was apparent in the measurements of the four biomarkers in the presence of other antibodies or serum proteins. These results supported the practical specificity of the Fc(IgG) and the PAD4, MCV, and CCP peptides employed for the recognition of the target antibodies, as well as the excellent selectivity of the amperometric transduction at the optimized working conditions.

### 3.4. Determination of RF, Anti-PAD4, Anti-MCV, and Anti-CCP in Human Serum

The simultaneous determination of the four RA biomarkers was accomplished in the human serum of patients diagnosed with RA, as well as in healthy individuals. The optimized procedure was applied to 25 µL aliquots of samples purchased from Central BioHub^®^. Firstly, the need for serum dilution was evaluated to fit the measured target concentrations to the respective calibration plot, as well as to avoid the matrix effects due to other endogenous components of the samples. The absence of matrix effects was verified by applying Student’s *t*-test to the comparison of the slope values of the calibration plots for the target standards in buffer solution with those obtained by applying the standard additions method, using a serum sample selected as a model (Ref. No 2998 BioHub^®^), containing certified concentrations of 118.9 IU mL^−1^ RF, 53.6 IU mL^−1^ anti-MCV, and 23.7 IU mL^−1^ anti-CCP. Supplementary Table S1 shows the obtained results for the direct analysis of raw serum in the cases of RF, anti-MCV and anti-CCP, as well as in the analysis of 1/1000 diluted serum for anti-PAD4. As can be seen, the t_exp_ values were, in all cases, lower than the tabulated t (t_tab_), therefore indicating that no apparent matrix effects occurred under the mentioned conditions. Therefore, the concentration of the endogenous antibodies in serum was quantified by interpolation of the responses measured for the samples into the calibration prepared with the target standards.

**Figure 4:**
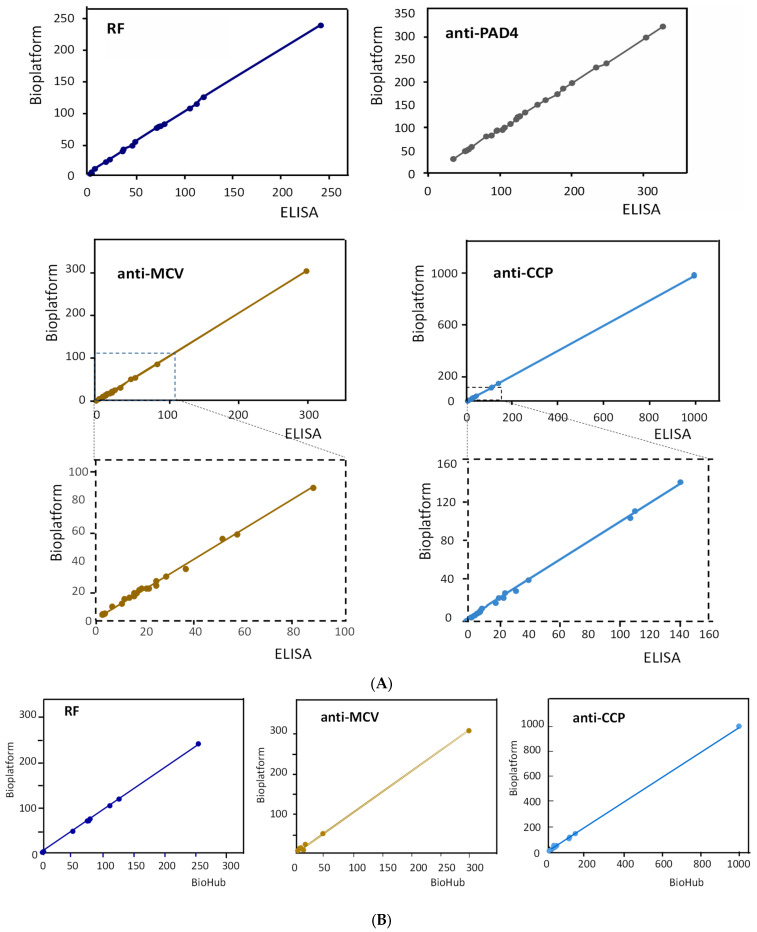
Correlation graphs of the results obtained for the determination of the target autoantibodies with the bioplatform and the individual ELISA kits (**A**) and the contents certified by Bio Hub^®^ (**B**).

The results obtained for the simultaneous amperometric determination of the four RA biomarkers in human serum are summarized in Table 3. Table 3 also includes the concentrations obtained by applying the individual ELISA kits and the certified contents provided by Central BioHub^®^ for RF, anti-MCV, and anti-CCP. As can be seen, a good agreement between the three sets of data was observed. The correlation studies shown in Figure 4A,B and summarized in Supplementary Table S2 show that the slope values of the correlation plots and the determination coefficients, R^2^, were practically equal to unity, and the intercept values were, in all cases, very close to zero. These results confirmed the excellent agreement between the results obtained with the quadruple bioplatform and those provided by the ELISA kits and the BioHub^®^ certified data.

Although no solid clinical conclusions can be drawn due to the relatively small size of the analyzed cohort of patients, some partial conclusions related to the contents of the biomarkers found in the serum samples and about the relationships between the different antibodies can be attained. The cutoff values for the target autoantibodies found in the literature, although with some variability, establish the following concentrations for serum-positive patients: RF > 20 IU mL^−1^ [23], anti-MCV > 20 IU mL^−1^ [24]; anti-PAD4 > 4749 IU mL^−1^ [25], and anti-CCP > 4.5 IU mL^−1^ [24]. In our case, the samples from healthy individuals provided concentrations of RF, anti-MCV, and anti-CCP that were lower than 4 IU mL^−1^ in all cases but between 35 and 60 kIU mL^−1^ of anti-PAD4. These latter values are much higher than the mentioned cutoff level. In addition, it can be observed that several serum samples from patients with concentrations of RF and/or anti-CCP below the cutoff values also exhibited high anti-PAD4 levels. Furthermore, more than half of these samples corresponded to patients treated with TNF inhibitors, as revealed by the information provided by the supplier (Central BioHub^®^, Hennigsdorf, Germany). These observations agree with those reported in the literature [19] because it is known that up to 60% of early RA patients may be RF and/or anti-CCP seronegative but seropositive for anti-PAD4, with this revealing the high diagnostic power of anti-PAD4 since it makes it possible to detect the disease in the absence of the most common biomarkers. Furthermore, the presence of anti-PAD4 is associated with higher disease severity, and its prevalence and resistance to treatment with TNF inhibitor drugs have also been claimed [26]. Regarding anti-MCV, no significant differences were found with respect to the concentrations of anti-CCP or R; moreover, single anti-MCV positivity has not been detected either. Therefore, similar to that indicated by other authors [6], this antibody does not seem to provide additional diagnostic information over the other common biomarkers.

## 4. Conclusions

The first electrochemical quadruple immunoplatform for the simultaneous determination of RF, anti-PAD4, anti-MCV, and anti-CCP antibodies—all of them relevant biomarkers of rheumatoid arthritis (RA)—is reported in this paper. The developed methodology relies on the implementation of sandwich-type bioassays between Fc(IgG) or the respective PAD4, MCV, or CCP antigens captured onto carboxylated MBs or onto neutravidin-functionalized MBs, and HRP-labeled detector antibodies by using quadruple SPC_4_Es as electrochemical support. The implemented method for the multiple determinations of the four biomarkers provides excellent analytical characteristics in terms of sensitivity, selectivity, and wide linear ranges and allows the accurate analysis to be performed in serum, without matrix effects, using 25 μL of raw or 1000-fold diluted sample. The developed methodology is competitive with commercial ELISA kits available only for the single determination of the biomarkers since the developed immunoplatform involves simple working protocols leading to shorter assay times and affordable cost. Indeed, the assay can be completed in two hours counting from the immobilization of the antigens on the functionalized MBs and using a sample volume as low as 25 μL for each target. This good performance, together with the high degree of multiplexing and the possibility that the determinations can be made in the field by any user, make the developed methodology suitable to meet the demands of current clinical practice to assist in the diagnosis of RA patients.

## Figures and Tables

**Table 1:** Experimental variables selected for the simultaneous determination of RF, anti-PAD4, anti-MCV, and anti-CCP with the developed quadruple immunoplatform.

Target Autoantibody	Variable	Tested Range	Selected Value
RF	Fc(IgG) loading, µg mL^−1^	5–35	25
	Incubation time Fc(IgG), min	15–60	30
	HRP-IgM, dilution	1/3; 1/2; no dilution	no dilution
	Incubation time HRP-IgM, min	30–90	60
Anti-PAD4	PAD4 loading, µg mL^−1^	5–20	15
	Incubation time PAD4, min	30–75	45
	Incubation time ethanolamine, min	15–35	30
	HRP-IgG dilution	1/5–1/20	1/10
	Incubation steps	1, 2	1
	Incubation time, anti-PAD4+HRP-IgG, min	30–75	45
Anti-MCV	MCV loading, µg mL^−1^	2–20	10
	Incubation time MCV, min	15–45	30
	HRP-IgG loading, µg mL^−1^	0.1–1	0.2
	Incubation time, HRP-IgG, min	15–45	30
	Incubation time ethanolamine, min	0–45	30
Anti-CCP	CCP-Biotin loading, µg mL^−1^	5–100	25
	Incubation time CCP-Biotin, min	15–90	30
	HRP-IgG dilution	1/4; 1/2; no dilution	no dilution
	Incubation time HRP-IgG, min	15–60	30

**Table 2:** Analytical characteristics of the calibration plots for RF, anti-PAD4, anti-MCV, and anti-CCP constructed with the quadruple immunoplatform.

Parameter	RF	Anti-PAD4	Anti-MCV	Anti-CCP
Slope	883 ± 21 nA/conc.decade (IU mL^−1^)	4.0 ± 0.1 nA/IU mL^−1^	12 ± 0.3 nA/ng mL^−1^	1348 ± 29 nA nA/conc.decade (IU mL^−1^)
Intercept	−191 ± 35 nA	253 ± 24 nA	435 ± 52 nA	−606 ± 59 nA
Linear range	3–300 IU mL^−1^	10–500 IU mL^−1^	1–300 ng mL^−1^	3–1000 IU mL^−1^
R^2^	0.998	0.998	0.998	0.999
LOD	1.0 IU mL^−1^	5.5 IU mL^−1^	0.2 ng mL^−1^	1.0 IU mL^−1^
LOQ	3.3 IU mL^−1^	18 IU mL^−1^	2.5 ng mL^−1^	3.2 IU mL^−1^
RSD, % (*n* = 10) (intra-day)	4.1 (0 IUmL^−1^) 4.3 (30 IUmL^−1^)	3.2 (0 IUmL^−1^) 3.8 (250 IUmL^−1^)	3.5 (0 ng mL^−1^) 4.3 (20 ng mL^−1^)	3.9 (0 IUmL^−1^) 4.4 (20 IUmL^−1^)
RSD, % (*n* = 10) (inter-day)	4.7 (0 IUmL^−1^) 5.0 (30 IUmL^−1^)	4.0 (0 IUmL^−1^) 4.6 (250 IUmL^−1^)	4.0 (0 ng mL^−1^) 4.7 (20 ng mL^−1^)	4.2 (0 IUmL^−1^) 4.6 (20 IUmL^−1^)

**Table 3:** Determination of the endogenous concentrations of the four autoantibodies in serum samples of healthy individuals and patients diagnosed with RA, with the quadruple bioplatform and ELISA, and certified concentrations from Central BioHub^®^ Product List.

			RF, IU mL^−1^			Anti-PAD, kIU mL^−1^		Anti-MCV, IU mL^−1^			Anti-CCP, IU mL^−1^		
	n	Reference *	Bioplatform	Bio Hub	ELISA	Bioplatform	ELISA	Bioplatform	Bio Hub	ELISA, IUmL^−1^	Bioplatform	Bio Hub	ELISA
Healthy individuals	1	8097	3.3 ± 0.3		3.2 ± 0.5	35 ± 5	34 ± 7	3.3 ± 0.3		3.2 ± 0.4	3.4 ± 0.6		3.1 ± 0.9
	2	8118	3.1 ± 0.4		3.1 ± 0.8	56 ± 10	55 ± 13	3 ± 1		2.9 ± 0.7	3.4 ± 0.5		3.3 ± 0.7
	3	8139	3.8 ± 0.3		3.9 ± 0.3	60 ± 7	60 ± 11	3 ± 1		2.6 ± 0.8	3.3 ± 0.5		3.2 ± 0.9
	4	8160	3.5 ± 0.2		3.5 ± 0.6	53 ± 13	52 ± 15	3.6 ± 0.9		3.4 ± 0.7	3.4 ± 0.2		3.4 ± 0.6
Patients diagnosed with RA	5	1169	3.1 ± 0.2		3.0 ± 0.4	244 ± 24	242 ± 38	17 ± 2		14 ± 4	3.2 ± 0.5	3.2	3.1 ± 0.8
	6	1623	7.7 ± 0.6		7.2 ± 0.9	121 ± 10	120 ± 20	14 ± 2	15	15 ± 4	7.6 ± 0.3	7.5	7.6 ± 0.6
	7	1723	3.9 ± 0.4		4.0 ± 0.5	177 ± 16	175 ± 28	11 ± 3		13 ± 3	107 ± 6	>100	105 ± 11
	8	1956	71 ± 5	71.8	72 ± 9	230 ± 16	233 ± 27	16 ± 4	15.8	16 ± 3	42 ± 2	44.3	43 ± 4
	9	2001	79 ± 6		78 ± 9	197 ± 13	199 ± 11	21 ± 5		20 ± 4	26 ± 8		30 ± 5
	10	2125	36 ± 3		35 ± 6	95 ± 7	95 ± 12	12 ± 2		10 ± 3	8 ± 4		7 ± 3
	11	2220	4.2 ± 0.4	4.8	4.1 ± 0.9	126 ± 14	125 ± 13	4 ± 1	3.1	3 ± 2	3.1 ± 0.8	1	3 ± 1
	12	2345	49 ± 5	49.8	50 ± 7	87 ± 10	85 ± 7	22 ± 2		20 ± 4	4.6 ± 0.2		4.6 ± 0.3
	13	2625	19 ± 4		18 ± 7	123 ± 10	126 ± 17	29 ± 2		28 ± 2	8.1 ± 0.9		9 ± 2
	14	2893	75 ± 9	75.8	75 ± 10	113 ± 8	110 ± 15	300 ± 14	307.6	300 ± 10	1000 ± 50	>1000	982 ± 68
	15	2998	119 ± 16	118.9	121 ± 21	80 ± 10	83 ± 12	52 ± 14	53.6	53 ± 16	23 ± 9	23.7	25 ± 11
	16	3019	37 ± 4		38 ± 6	51 ± 11	51 ± 14	37 ± 5		33 ± 4	7 ± 2		8 ± 4
	17	3084	239 ± 20	238.4	235 ± 27	133 ± 11	135 ± 16	58 ± 7		56 ± 8	34 ± 9	32.6	32 ± 12
	18	3973	23 ± 3		22 ± 8	150 ± 10	152 ± 11	25 ± 3		25 ± 2	1000 ± 54	>1000	993 ± 91
	19	4020	3.3 ± 0.4	3.4	3.2 ± 0.9	105 ± 8	102 ± 9	16 ± 2		17 ± 4	6.3 ± 0.3		6.6 ± 0.8
	20	4047	3.2 ± 0.4		3.3 ± 0.7	94 ± 11	96 ± 13	3.1 ± 0.8	2.3	3 ± 1	3.5 ± 0.3	3.75	3.4 ± 0.5
	21	4285	73 ± 7	72	74 ± 5	102 ± 16	97 ± 27	7 ± 1		8 ± 2	139 ± 23	140.5	141 ± 36
	22	4576	3.0 ± 0.3	3	3.1 ± 0.6	125 ± 10	127 ± 22	19 ± 4		20 ± 2	9 ± 2		11 ± 4
	23	4987	105 ± 15	104.8	103 ± 17	185 ± 17	187 ± 29	18 ± 3		19 ± 2	21 ± 7		20 ± 11
	24	5650	112 ± 9		110 ± 11	161 ± 18	162 ± 16	89 ± 3		87 ± 4	110 ± 15	112	112 ± 18
	25	6499	46 ± 3		44 ± 6	298 ± 14	298 ± 29	25 ± 3		22 ± 5	27 ± 3	47	25 ± 6
	26	8698	5.0 ± 0.4	5.4	4 ± 1	321 ± 16	322 ± 20	16 ± 4		17 ± 7	5.3 ± 0.5		5.0 ± 0.9

$\bar{x}\pm ts/\surd n\;\left(n=6,\;\mathrm{immunoplatform};n=4,\;\mathrm{ELISA};\;\alpha =0.05\right)\;$ * Reference numbers from the Product List of Central BioHub^®^.

## Data Availability

Not applicable.

## Supplementary Materials

The following supporting information can be downloaded at https://www.mdpi.com/article/10.3390/bios13030381/s1. Figure S1: Optimization of the experimental variables involved in the preparation and functioning of the immunoplatform constructed for the single determination of RF: (a) Fc(IgG) concentration; (b) incubation time of Fc(IgG), (c) HRP-IgM dilution and (d) incubation time of HRP-IgM. Amperometric responses measured in the absence (N; light bars) and in the presence of 300 IU mL-1 RF standard solutions (S; dark bars) as well as the resulting S/N ratio values (circles and black line); Figure S2: Optimization of the experimental variables involved in the preparation and functioning of the immunoplatform constructed for the single determination of anti-PAD4: (a) PAD4 concentration; (b) incubation time of PAD4, (c) incubation time with ethanolamine blocker; (d) HRP-IgG dilution; (e) number of incubation steps; (f) incubation time for the one-step protocol. Amperometric responses measured in the absence (N; light bars) and in the presence of 250 IU mL^−1^ anti-PAD standard solutions (S; dark bars) as well as the resulting S/N ratio values (circles and black line); Figure S3: Optimization of the experimental variables involved in the preparation and functioning of the immuno-platform constructed for the single determination of anti-MCV: (a) MCV concentration; (b) incubation time of MCV, (c) HRP-IgG concentration; (d) incubation time of HRP-IgG; (e) incubation time with the ethanolamine blocker. Amperometric responses measured in the absence (N; light bars) and in the presence of 20 ng mL^−1^ anti-MCV standard solutions (S; dark bars) as well as the resulting S/N ratio values (circles and black line); Figure S4: Optimization of the experimental variables involved in the preparation and functioning of the immunoplatform constructed for the single determination of anti-CCP: (a) CCP concentration; (b) incubation time of CCP, (c) HRP-IgG concentration; (d) incubation time of HRP-IgG. Amperometric responses measured in the absence (N; light bars) and in the presence of 20 IU mL^−1^ anti-CCP standard solutions (S; dark bars) as well as the resulting S/N ratio values (circles and black line); Figure S5: Control charts constructed to check the storage stability of: (a) Fc(IgG)-cMBs, (b) PAD-4-cMBs, (c) MCV-cMBs, and CCP-Biotin-Neutr-MBs bioconjugates at 8 °C in PBS (pH 7.2). Control limits (dashed lines) were set as ±2 s of the average amperometric current of three measurements obtained the day of bioconjugates preparation; Table S1: Comparison of the slope values of the calibration plots for target antibodies; Table S2: Correlation data for the results shown in Figure 3a,b.

## Abbreviation

List of abbreviations and acronyms

ACPAs	anti-citrullinated protein antibodies
anti-CCP	anti-cyclic citrullinated peptide antibody
anti-MCV	anti-citrullinated vimentin antibody
anti-PAD4	anti-peptidyl-arginine deiminase enzyme antibody
CXCL4	chemokine (C-X-C motif) ligand 4
CXCL7	chemokine (C-X-C motif) ligand 7
HB	hemoglobin
hIgG	human immunoglobulin G
HQ	hydroquinone
HRP	horseradish peroxidase
has	human serum albumin
IFN-γ	interferon-γ
IL-1β	interleukin-1β
IL-6	interleukin-6
MBs	magnetic microbeads
PB	phosphate buffer
RA	rheumatoid arthritis
RANKL	receptor activator of nuclear factor kappa-Β ligand
RF	rheumatoid factor
TMB	3,3′,5,5′-tetramethylbenzidine
TNF-α	tumor necrosis factor alpha
