# Supplementary material for: Serum Autoantibody Biomarkers for Management of Rheumatoid Arthritis Disease
Source: Biosensors (Basel). 2023 Mar 13;13(3):381. doi: 10.3390/bios13030381 (PMC10046368; doi:10.3390/bios13030381)
Supplement: Supplementary file 1 [file biosensors-13-00381-s001.zip › biosensors-2251293-supplementary.pdf]

SUPPLEMENTARY INFORMATION

Serum Autoantibody Biomarkers for Management of Rheumatoid Arthritis Disease

Esther Sánchez-Tirado [1], Lourdes Agüí [1], Marta Sánchez-Paniagua [2], Araceli González-Cortés[1], Beatriz López-Ruiz[2], Paloma Yáñez-Sedeño[1,*] and José M. Pingarrón[1]

[1] Department of Analytical Chemistry, Faculty of Chemistry, University Complutense of Madrid, 28040 Madrid, Spain

[2] Department of Chemistry in Pharmaceutical Sciences, Faculty of Pharmacy, University Complutense of Madrid, 28040 Madrid, Spain

* Correspondence: yseo@quim.ucm.es

1. Preparation of the magnetic immunoconjugates

To a 1.5 mL eppendorf tube, a 3 μL-aliquot of the functionalized MBs (cMBs for RF, anti-PAD4 and anti-MCV, and Neutr-MBs for anti-CCP) suspension was transferred. Then, two washings were made with 50 μL of 25 mM MES buffer solution of pH 5.0 (RF, anti-PAD4 and anti-MCV) or PBST of pH 7.2 (anti-CCP) by incubating during 10 minutes at 25 °C with constant stirring at 950 rpm. Each washing was carried out by placing the tube in the magnetic concentrator for 2 min and further removing of the supernatant. Next, 25 μL of a solution containing 50 mg mL^{-1} EDC and NHSS were added (for RF, anti-MCV and anti-PAD4) to activate the surface carboxylic groups, allowing incubation for 30 minutes at 37 °C (RF and anti-MCV) or at 25 ºC (anti-PAD4) with constant stirring. Thereafter, two washings with 50 μL of 25 mM MES buffer solution of pH 5.0 were made.

The immunoconjugates for the determination of RF were prepared by addition of 25 μL of a 25 μg mL^{-1} Fc (IgG) solution in 25 mM MES buffer solution of pH 5.0 to the activated cMBs, allowing incubation for 30 min at 37 ºC. Then, the biofunctionalized MBs were separated, the supernatant discarded, and two washings with 50 μL of 25 mM MES buffer solution of pH 5.0 were carried out. Next, 25 μL of the antibody (RF) solution (or the serum sample) were added and incubated for 60 minutes at 37 °C with constant stirring at 950 rpm. The resulting RF-Fc(IgG)-cMBs particles were separated using the magnetic separator, the supernatant discarded, and two washings were performed with 50 μL of washing buffer (WB) from the RF ELISA kit AB178653 (Abcam). Thereafter, 25 μL of the commercial HRP-IgM conjugate were added and incubated for 60 minutes at 37°C with constant stirring at 950 rpm. Finally, once the HRP-IgM-RF-Fc(IgG)-cMBs were magnetically separated, two washings were performed with 50 μL of WB.

The anti-PAD4 or anti-MCV immunoconjugates were prepared by addition of 25 μL of 15 μg mL^{-1} PAD4 or 10 μg mL^{-1} MCV solutions in 25 mM MES buffer of pH 5.0 to the activated cMBs, followed by addition of 25 μL of 1M ethanolamine in 100 mM PB of pH 8.0, and allowing incubation for 30 min at 25 ºC (PAD4) or at 37 ºC (MCV). Then, the PAD4-cMBs or MCV-cMBs were separated, the supernatants discarded, and two washings with 50 μL of WB from the anti-PAD4 ELISA kit 500,930 (Cayman) or the anti-MCV ELISA kit MBS9716536 (MyBiosource), respectively, were made. The preparation of the anti-PAD4 immunoconjugate involved the addition of 25 μL of a mixture solution of anti-PAD4 standards (or the sample) and 1/10 diluted HRP-anti-IgG to the PAD4-cMBs and incubation for 45 min at 25 ºC to form the HRP-anti-IgG-anti-PAD4-PAD4-cMBs immunoconjugates. Finally, two washings with the corresponding WB were performed. In the case of anti-MCV, 25 μL of anti-MCV standards (or the sample) were added and incubated for 30 min at 37 ºC followed by two washings with 50 μL of the corresponding WB. Thereafter, 25 μL of 0.2 μg mL^{-1} HRP-anti-IgG prepared in BB were added and incubated for 30 min at 37 ºC. Finally, the HRP-anti-IgG-anti-MCV-MCV-cMBs were separated, the supernatants discarded, and two washings with 50 μL of the corresponding WB were made.

The preparation of the anti-CCP immunoconjugates was accomplished by incubation of the Neutr-MBs with 25 µL of CCP-Biotin in 100 mM PBS of pH 7.2 for 30 min at 25 ºC. After two washings with 50 µL PBST, the supernatant was removed and 25 µL of anti-CCP standard solution (or the serum sample) were incubated with the CCP-Biotin-Neutr-MBs during 60 min at 25 ºC and washed twice as mentioned above. Thereafter, the modified MBs were incubated with 25 µL of HRP-IgG solution for 45 min at 25 ºC and the resulting HRP-IgG-CCPA-CCP-Biotin-Neutr-MBs bio-conjugates were washed twice as indicated above.

2. Optimization of variables involved in the preparation of the immunoplatform for the simultaneous determination of RF, anti-PAD4, anti-MCV and anti-CCP

The results obtained in the optimization of the experimental variables involved in the functioning of the immunoplatform constructed for the single amperometric detection of RF is displayed in **Figure S1**. The effect of the Fc(IgG) concentration, over the 5 to 500 µg mL^{-1} range, and the incubation time for immobilization on activated cMBs, between 15 and 60 min at 37 ºC, 950 rpm, were tested in the absence (N) and in the presence (S) of 300 IU mL^{-1} RF. **Figures S1a** and **S1b** show as a Fc(IgG) concentration smaller than 25 µg mL^{-1} gave rise to lower amperometric responses due to the limited loading of capture antigen. Moreover, the S/N ratio decreased for larger concentrations probably because of the steric hindrance recognition. Therefore, a Fc(IgG) concentration of 25 µg mL^{-1} was chosen for further work. Regarding the incubation time, a better S/N ratio was reached at 30 min. It is important to note that at these selected experimental conditions, the non-specific current (N) was of only 0.037 ± 0.013 µA vs. 1.44 ± 0.07 µA measured for 300 IU mL^{-1} (S), therefore demonstrating the successful design of the sandwich-format immunoassay and the practical absence of unspecific adsorptions with no need for blocking.

Regarding the concentration and incubation time of the HRP-IgM detection antibody, **Figures S1c** and **S1d** show the results obtained by incubating the RF-Fc(IgG)-cMBs conjugates in undiluted, 1/3 and 1/2 diluted HRP-IgM solutions over the 30 to 90 min period. Interestingly, a larger S/N ratio~~s~~ was found for undiluted HRP-IgM solution and 60 min since smaller HRP-IgM concentrations and shorter incubation times provoked a large decrease of the specific responses. Therefore, the above mentioned values were selected for the preparation of the immunoplatform.

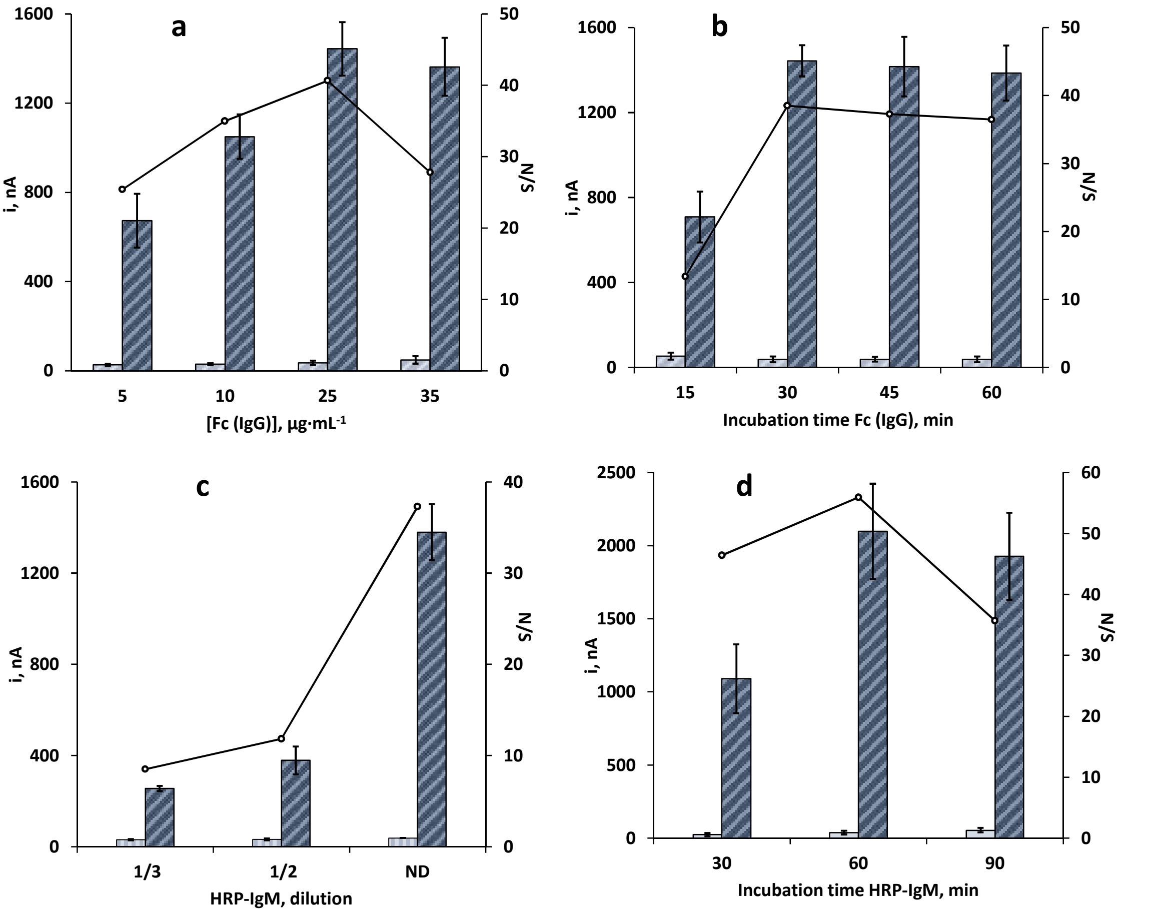

Figure S1. Optimization of the experimental variables involved in the preparation and functioning of the immunoplatform constructed for the single determination of RF: a) Fc(IgG) concentration; b) incubation time of Fc(IgG), c) HRP-IgM dilution and d) incubation time of HRP-IgM. Amperometric responses measured in the absence (N; light bars) and in the presence of 300 IU mL^{-1} RF standard solutions (S; dark bars) as well as the resulting S/N ratio values (circles and black line).

Figure S2. shows the results obtained for the optimization of the experimental variables involved in the performance of the immunoplatform prepared for the single amperometric determination of anti-PAD 4. The effect of the concentration of PAD4 peptide immobilized onto activated cMBs on the measurements carried out in the absence (N) and in the presence (S) of 250 IU mL^{-1} anti-PAD4 (**Figure S2a**) was tested over the 5 to 20 µg mL^{-1} range incubating at 25 ºC for 60 min. As it can be seen, a better S/N ratio was found for 15 µg mL^{-1} antigen. Smaller concentrations gave rise to lower amperometric currents as a consequence of the insufficient peptide loading thus resulting in smaller S/N ratios. Concentrations larger than 15 µg mL^{-1} gave also rise to a decrease of the S/N ratio probably due to hindered recognition for the target antibody similarly to that observed for Fc(IgG) (**Figure S1a**). Regarding the incubation time for PAD4, the results shown in **Figure S2b** allowed 45 min to be selected since shorter times provoked a decrease of the S/N ratio due to the smaller efficiency for the immunorecognition process. With the aim of minimizing non-specific adsorptions through the residual activated carboxylic groups, the PAD4-cMBs conjugates were incubated in 1M ethanolamine used as the blocker solution. The incubation time was checked over the 15 to 45 min range and **Figure S2c** shows as a better S/N ratio was obtained for 30 min. The effect of the HRP-IgG concentration on the amperometric responses was also evaluated using dilutions between 1/20 and 1/5 (**Figure S2d**). Larger S/N values were obtained when working with the 1/10 dilution probably because more diluted solutions resulted in a limiting concentration of enzymatic tracer, worsening discrimination.

In addition, different working protocols were tested as it is illustrated in **Figure S2e.** The protocols started from the prepared anti-PAD4-PAD4-cMBs and involved 60 min incubation steps at 25 ºC, 950 rpm: A) a single step through the incubation with a mixture solution containing 0 or 250 IU mL^{-1} anti-PAD4 standard and 1/10 diluted HRP-IgG, and B) two successive incubation steps with 0 or 250 IU mL^{-1} anti-PAD4 standard and 1/10 diluted HRP-IgG. As it can be seen, a larger S/B ratio was obtained by applying the protocol A, probably due to the improved immunorecognition efficiency and labelling reactions when all immunoreagents were in homogeneous solution. Additionally, this protocol has the advantage of the shortening and simplification of the immunoassay procedure. Finally, the time for incubation of the mixture solution containing anti-PAD4 and HRP-IgG was evaluated with the results shown in **Figure S2f**. As it can be observed, a better S/N ratio was obtained for 45 min.

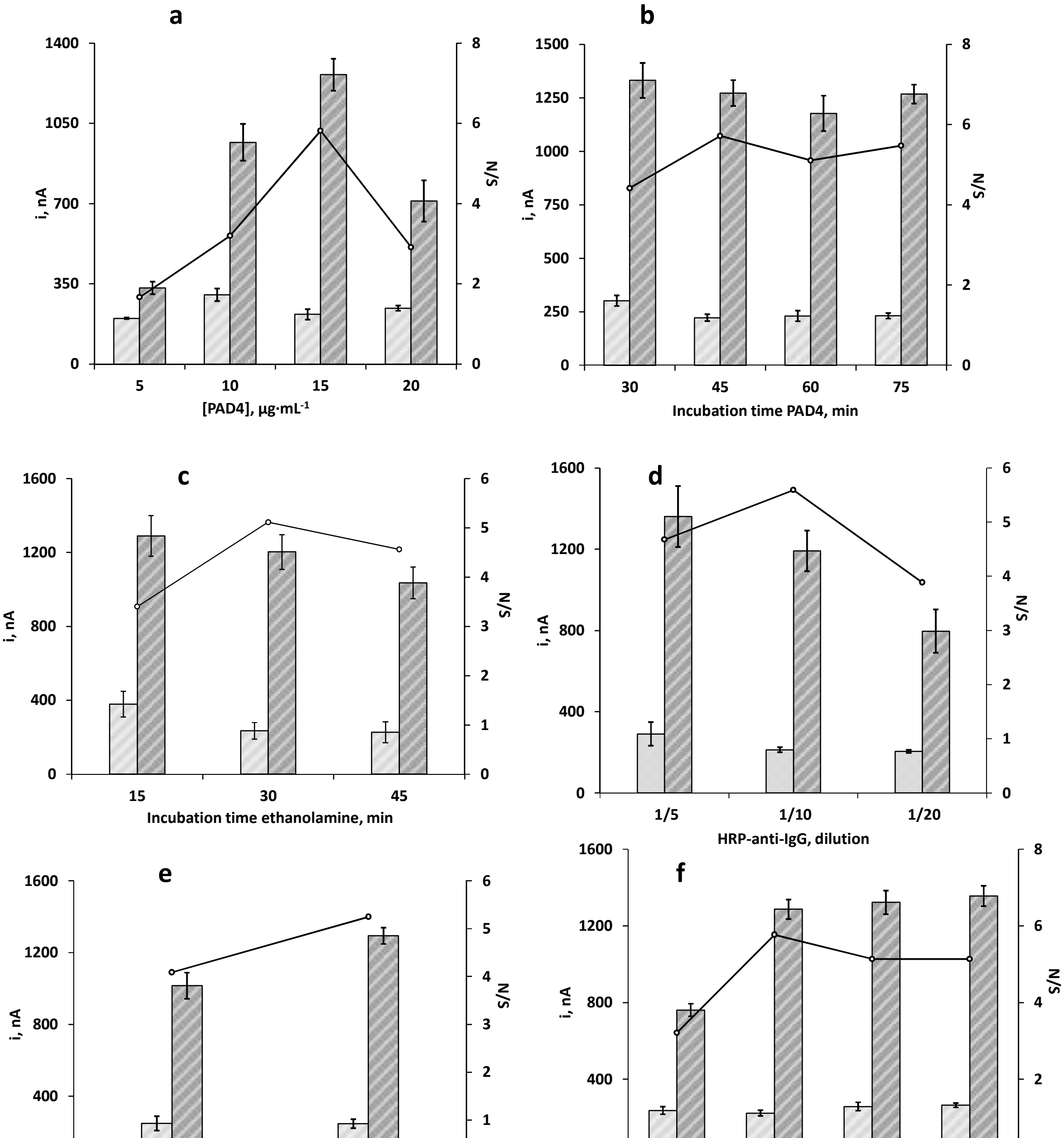

Figure S2. Optimization of the experimental variables involved in the preparation and functioning of the immunoplatform constructed for the single determination of anti-PAD4: a) PAD4 concentra-

tion; b) incubation time of PAD4, c) incubation time with ethanolamine blocker; d) HRP-IgG dilution; e) number of incubation steps; f) incubation time for the one-step protocol. Amperometric responses measured in the absence (N; light bars) and in the presence of 250 IU mL^{-1} anti-PAD standard solutions (S; dark bars) as well as the resulting S/N ratio values (circles and black line).

The results obtained in the optimization of variables involved in the preparation and functioning of the immunoplatform constructed for the single determination of anti-MCV are shown in **Figure S3.** The effect of the MCV concentration immobilized onto the activated cMBs (**Figure S3a**) was studied in the range between 2 and 20 μg mL^{-1} in the absence (N) and in the presence (S) of 20 ng mL^{-1} anti-MCV incubated at 37 ºC for 30 min. A better S/N ratio was found for 10 μg mL^{-1}. Lower MCV concentrations produced a dramatic decrease of the specific currents along with an increase in non-specific responses probably due to an insufficient peptide loading thus leading to remarkably smaller S/N ratios. Furthermore, concentrations larger than 10 μg mL^{-1} gave rise to smaller S/N ratios as a result of the probable hindered recognition for the target antibody. Regarding the incubation time for MCV (**Figure S3b**), 30 min was selected, since longer times provided lower S/N ratios due to the loss of efficiency for the immunorecognition process.

The effect of the HRP-IgG loading was checked between 0.1 and 1 μg mL^{-1} (**Figure S3c**). The specific responses remained practically constant from 0.2 μg mL^{-1} most likely because smaller concentrations did not allow a good discrimination. However, the increase in the enzymatic conjugate provoked an increase of the non-specific responses leading to a rapid decrease of the S/N ratio. Therefore, 0.2 μg mL^{-1} was selected for the preparation of the immunoplatform. Regarding the incubation time of HRP-IgG, the results displayed in **Figure S3d,** led us to select 30 min for further work. In addition, the effect of the incubation time with 1M ethanolamine to block the residual activated carboxylic groups of PAD4-cMBs is shown in **Figure S3e**. As expected, the non-specific responses decreased as increasing

the incubation time with the blocker solution, whilst the specific currents remained almost constant up to 30 min.

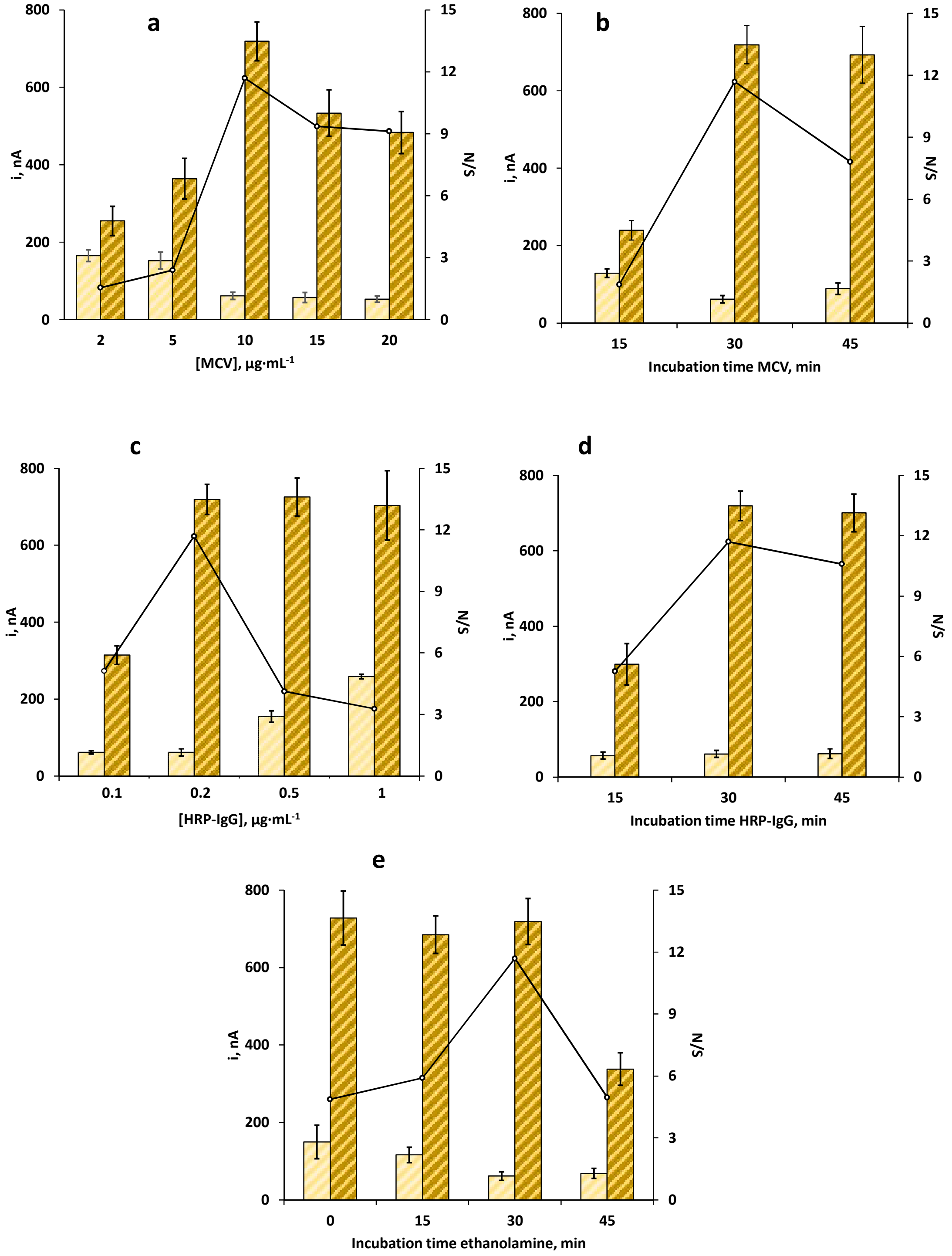

Figure S3. Optimization of the experimental variables involved in the preparation and functioning of the immunoplatform constructed for the single determination of anti-MCV: a) MCV concentration; b) incubation time of MCV, c) HRP-IgG concentration; d) incubation time of HRP-IgG; e) incubation time with the ethanolamine blocker. Amperometric responses measured in the absence (N; light bars) and in the presence of 20 ng mL^{-1} anti-MCV standard solutions (S; dark bars) as well as the resulting S/N ratio values (circles and black line).

The experimental variables involved in the preparation and functioning of the immnoplatform constructed for the individual determination of anti-CCP were optimized by measuring the amperometric currents in the absence (N) and in the presence (S) of 20 IU mL^{-1} anti-CCP. The effect of CCP-Biotin loading was checked between 5 and 100 µg mL^{-1} upon incubation during 60 min at 25 ºC. Also, the incubation time with the Neutr-

MBs was tested in the 15 to 90 min range. **Figures S4a** and **S4b** show as the specific currents remained practically constant from 25 µg mL^{-1} CCP-Biotin whereas smaller concentrations gave rise to lower specific currents since there was not enough amount of antigen to capture the target antibody. In addition, an incubation time of 30 min yielded a larger S/N ratio and thus, these values were selected for further work. The effect of the HRP-IgG dilution on the amperometric response is shown in **Figure S4c.** The specific currents increased as the dilution factor decreased while the nonspecific current kept practically constant. Therefore, a better S/N ratio was obtained when using undiluted conjugate solution. Moreover, 30 min showed to be an adequate incubation time of HRP-IgG with anti-CCP-CCP-Biotin-Neutr-MBs.

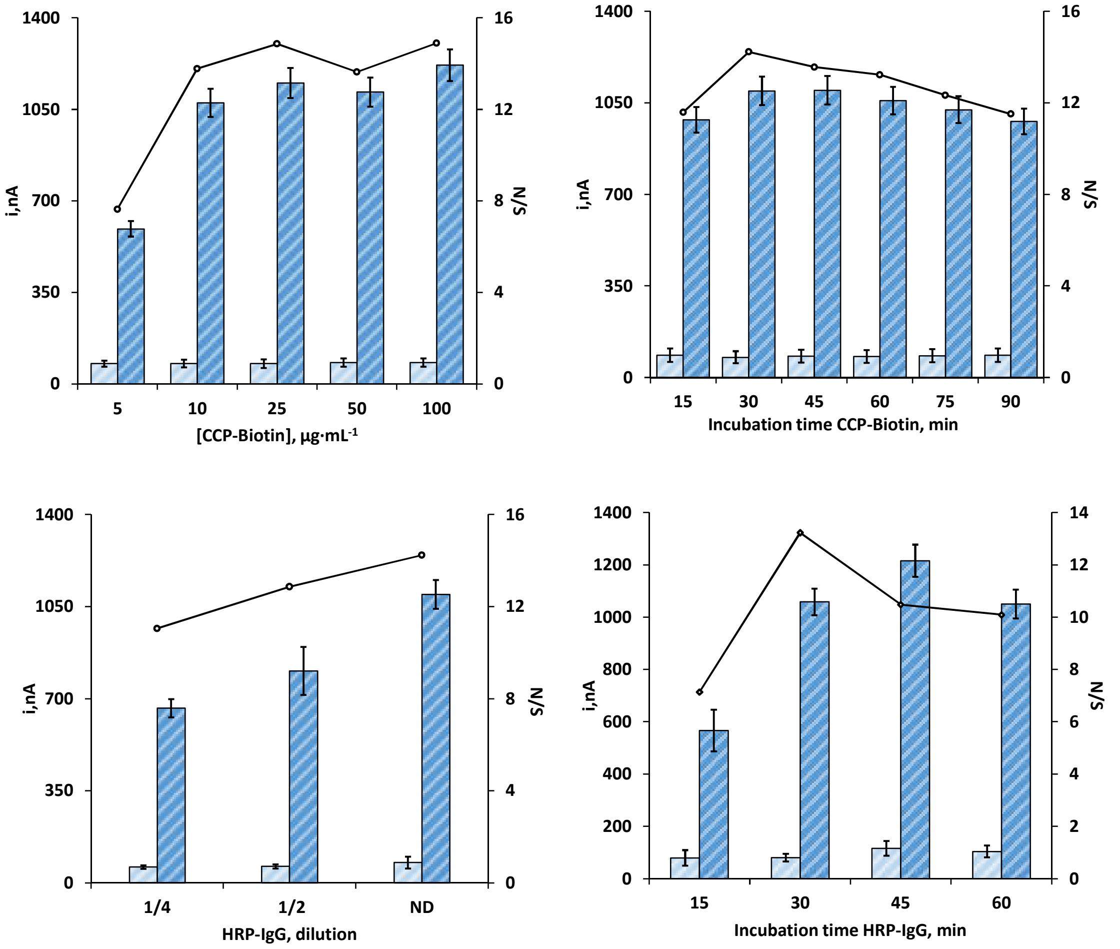

Figure S4. Optimization of the experimental variables involved in the preparation and functioning of the immunoplatform constructed for the single determination of anti-CCP: a) CCP concentration; b) incubation time of CCP, c) HRP-IgG concentration; d) incubation time of HRP-IgG. Amperometric responses measured in the absence (N; light bars) and in the presence of 20 IU mL^{-1} anti-CCP standard solutions (S; dark bars) as well as the resulting S/N ratio values (circles and black line).

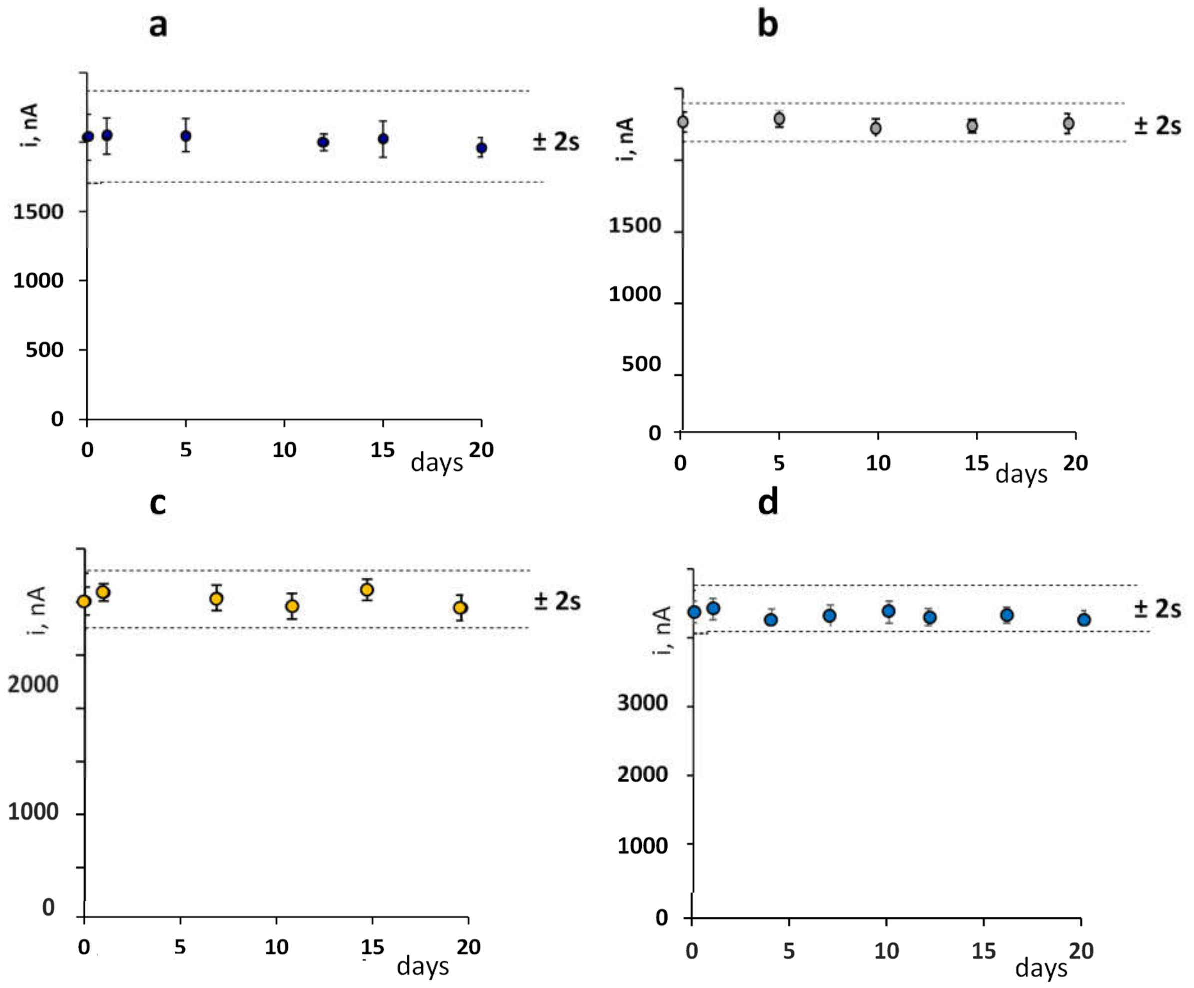

Figure S5. Control charts constructed to check the storage stability of: a) Fc(IgG)-cMBs, b) PAD-4-cMBs, c) MCV-cMBs, and CCP-Biotin-Neutr-MBs bioconjugates at 8 ºC in PBS (pH 7.2). Control limits (dashed lines) were set as ±2s of the average amperometric current of three measurements obtained the day of bioconjugates preparation.

Table S1. Comparison of the slope values of the calibration plots for target antibodies.

	**Calibration plot for standards in PB pH 6.0**	**Standard additions in serum***	
**Target**	**Slope**	**Slope**	**t_{exp} (t_{tab})**
**RF[a]**	883 ± 21 nA/conc. decade IU mL^{-1}	680 ± 99 nA/conc. decade IU mL^{-1}	2.077 (2.447) (n=6)
**anti-PAD4[b]**	3.98 ± 0.09 nA/IU mL^{-1}	3.70 ± 0.08 nA/IU mL^{-1}	1.857 (2.447) (n=6)
**anti-MCV[a]**	12.0 ± 0.3 ng mL^{-1}	13 ± 0.4 ng mL^{-1}	1.767 (2.447) (n=6)
**anti-CCP[a]**	1348 ± 29 nA/conc. decade IU mL^{-1}	1248 ± 29 nA/conc. decade IU mL^{-1}	2.059 (2.447) (n=6)

*Sample Ref No 2998; a) undiluted serum; b) 1/1000 diluted serum.

Table S2. Correlation data for the results shown in Figures 3A and B.

**Biomarker**	**Immunoplatform vs. ELISA**			**Immunoplatform vs. Bio Hub**		
	**Slope**	**Intercept**	**R^2**	**Slope**	**Intercept**	**R^2**
**RF**	0.999 ± 0.004	+0.1 ± 0.3	0.9996	0.996 ± 0.003	+0.4 ± 0.2	0.9999

**anti-PAD4**	1.006 ± 0.005	-0.8 ± 0.8	0.9993	-	-	-
**anti-MCV**	0.999 ± 0.001	-0.5 ± 0.3	0.9995	1.037 ± 0.003	-0.4 ± 0.3	0.9999
**anti-CCP**	0.99 ± 0.01	+0.5 ± 0.4	0.9999	0.998 ± 0.005	+1 ± 2	0.9997

List of abbreviations and acronyms

ACPAs: anti-citrullinated protein antibodies
anti-CCP: anti-cyclic citrullinated peptide antibody
anti-MCV: anti-citrullinated vimentin antibody
anti-PAD4: anti-peptidyl-arginine deiminase enzyme antibody
CXCL4: chemokine (C-X-C motif) ligand 4
CXCL7: chemokine (C-X-C motif) ligand 7
HB: hemoglobin
hIgG: human immunoglobulin G
HQ: hydroquinone
HRP: horseraddish peroxidase
HSA: human serum abumin
IFN-γ: interferon-γ
IL-1β: interleukin-1β
IL-6: interleukin-6
MBs: magnetic microbeads
PB: phosphate buffer
RA: rheumatoid arthritis
RANKL: receptor activator of nuclear factor kappa-B ligand
RF: rheumatoid factor
TMB: 3,3′,5,5′-tetramethylbenzidine
TNF-α: tumor necrosis factor alpha
